# Risk factors for diagnostic delay in idiopathic pulmonary fibrosis

**DOI:** 10.1186/s12931-019-1076-0

**Published:** 2019-05-24

**Authors:** Nils Hoyer, Thomas Skovhus Prior, Elisabeth Bendstrup, Torgny Wilcke, Saher Burhan Shaker

**Affiliations:** 1 Department of Respiratory Medicine, Herlev and Gentofte Hospital, Kildegårdsvej 28, 2900 Hellerup, Denmark; 2 Department of Respiratory Diseases and Allergy, Aarhus University Hospital, Nørrebrogade 44, 8000 Aarhus C, Denmark

**Keywords:** IPF, Diagnosis, Delay, Cohort, Observational

## Abstract

**Background:**

Surveys and retrospective studies of patients with idiopathic pulmonary fibrosis (IPF) have shown a significant diagnostic delay. However, the causes and risk factors for this delay are not known.

**Methods:**

Dates at six time points before the IPF diagnosis (onset of symptoms, first contact to a general practitioner, first hospital contact, referral to an interstitial lung disease (ILD) centre, first visit at an ILD centre, and final diagnosis) were recorded in a multicentre cohort of 204 incident IPF patients. Based on these dates, the delay was divided into specific patient-related and healthcare-related delays. Demographic and clinical data were used to determine risk factors for a prolonged delay, using multivariate negative binomial regression analysis.

**Results:**

The median diagnostic delay was 2.1 years (IQR: 0.9–5.0), mainly attributable to the patients, general practitioners and community hospitals. Male sex was a risk factor for patient delay (IRR: 3.84, 95% CI: 1.17–11.36, *p* = 0.006) and old age was a risk factor for healthcare delay (IRR: 1.03, 95% CI: 1.01–1.06, *p* = 0.004). The total delay was prolonged in previous users of inhalation therapy (IRR: 1.99, 95% CI: 1.40–2.88, *p* <  0.0001) but not in patients with airway obstruction. Misdiagnosis of respiratory symptoms was reported by 41% of all patients.

**Conclusion:**

Despite increased awareness of IPF, the diagnostic delay is still 2.1 years. Male sex, older age and treatment attempts for alternative diagnoses are risk factors for a delayed diagnosis of IPF. Efforts to reduce the diagnostic delay should focus on these risk factors.

**Trial registration:**

This study was registered at http://clinicaltrials.gov (NCT02772549) on May 10, 2016.

**Electronic supplementary material:**

The online version of this article (10.1186/s12931-019-1076-0) contains supplementary material, which is available to authorized users.

## Background

Idiopathic Pulmonary Fibrosis (IPF) is a debilitating chronic lung disease. Despite recent advances in antifibrotic therapy, the prognosis is poor with a median survival of 2–5 years [1, 2]. The diagnosis of IPF can be challenging, and due to its rarity, few physicians gain enough expertise to become familiar with the disease. In addition, the diagnosis often requires a multidisciplinary team, which is not available in all centres [3, 4]. Surveys and retrospective studies have shown a significant diagnostic delay from the onset of symptoms until the final diagnosis is made [5–8]. This results in a delayed start of effective antifibrotic treatment and lung transplant evaluation, and can affect the disease course and prognosis [5]. Current medical treatment of IPF can slow down the progression of the disease but does not seem to reverse any fibrotic changes that already have occurred [9–11]. Timely diagnosis is therefore important in order to initiate early treatment, prevent lung tissue from developing fibrosis, delay disease progression and prolong survival [4, 8].

The cause of the diagnostic delay is not well known but is probably multifactorial. Surveys suggest that misinterpretation of respiratory symptoms could be a contributing factor [6, 12]. The first symptoms of IPF are often cough and shortness of breath, which are often attributed to old age, smoking or more common cardiovascular or respiratory diseases by either the patient or healthcare professionals as it is the case in patients with chronic obstructive pulmonary disease (COPD) [13]. The distribution of the diagnostic delay between patient-related and healthcare-related causes is still largely unknown. Moreover, we are not aware of any studies detailing the risk factors for a delayed diagnosis. A thorough understanding of these risk factors is essential for any attempts to reduce the diagnostic delay.

To answer these questions, we used a well characterized multicentre cohort of incident IPF patients to investigate the diagnostic delay and determine specific risk factors for a delayed diagnosis.

## Materials and methods

### Patient cohort

The Pulmonary Fibrosis Biomarker (PFBIO) cohort prospectively recruits incident patients with IPF from two large interstitial lung disease (ILD) centres in Denmark serving a population of 4.2 million. The main purpose of the cohort is to follow a broad population of patients with IPF and create a biorepository for biomarker research. Antifibrotic therapy is centralized in Denmark and referral to one of the three specialized ILD centres is necessary for patients to be eligible for treatment. Recruitment for the PFBIO cohort started in 2016 and is still ongoing at one of the participating centres (Gentofte Hospital) for future studies. At the time of analysis, the cohort had included 204 patients. All participants had received a diagnosis of IPF according to the 2011 ATS/ERS/JRS/ALAT guidelines at an ILD centre, based on a multidisciplinary team approach [3, 14]. Patients were recruited on the same day as they received the diagnosis or up to a maximum of two months later but prior to initiating antifibrotic treatment. All patients with IPF were eligible for participation unless they were unable to provide written informed consent. A flowchart of the recruitment process is presented in the Additional files 1.

### Data collection

Patients completed a detailed survey at the time of diagnosis which was followed up by an interview if needed for clarification (see Additional file 1). The survey included questions about the onset of symptoms, initial healthcare contact, smoking history, alternative diagnoses for respiratory symptoms, and previous therapies. The patients’ quality of life was assessed by the St. George’s Respiratory Questionnaire (SGRQ) with higher scores indicating worse quality of life [15]. Several measures of disease severity were collected from the patients’ electronic records: comprehensive pulmonary function tests with spirometry and diffusing capacity for carbon monoxide (DLCO), six-minute walk test (6MWT) and high resolution computed tomography (HRCT) scan.

**Fig. 1:**
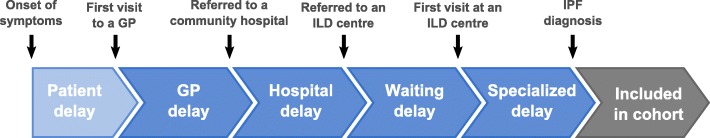
Diagnostic delay from patients’ awareness of symptoms until an IPF diagnosis is made. The total delay is divided into patient delay and healthcare delay (GP delay, hospital delay, waiting delay and specialized delay combined)

The diagnostic delay was calculated based on six pre-defined time points on the patients’ path towards an IPF diagnosis (Fig. 1). The first two time points (dates of symptom onset and first contact with a general practitioner) were part of the survey which was completed by each patient shortly after their diagnosis. All remaining time points were collected from the patients’ electronic records. The specific delays were calculated from these time points and are illustrated in Fig. 1. If a time point was not relevant in a patient’s path towards an IPF diagnosis (i.e. if the first contact with a physician was at a community hospital or if a patient was referred directly to an ILD centre by the general practitioner), the jumped delay was excluded for that patient in the statistical analysis.

### Statistical analysis

Descriptive data are presented as frequency tables, mean (SD) or median (interquartile range, IQR) as appropriate for the data. Univariate analyses of baseline characteristics were performed with a t-test or chi-squared test as appropriate for the data.

None of the specific delays were normally distributed and are presented as medians with IQR. We used multivariate negative binomial regression models to determine specific risk factors for patient delay, healthcare delay and total delay respectively, as the delays were counts of days and did not fit a Poisson distribution.

Covariates were tested for normality before inclusion into any statistical analysis. Missing data were handled by listwise deletion using complete cases without any imputation of data.

### Ethics, consent and permissions

All participants were included after written informed consent, and none had chosen to withdraw from the study at the time of analysis. The study has been approved by the Capital Region regional ethics committee (H-16001790) and the Danish Data Protection Agency (HGH-2016-017). The study was also registered at http://clinicaltrials.gov (NCT02772549). We conducted the study according to the principles of the Declaration of Helsinki and adhered to the STROBE guidelines.

## Results

The baseline characteristics of the 204 participants in the PFBIO cohort are listed in Table 1. Patients were stratified according to length of total diagnostic delay above or below 2 years. Demographic data such as age and sex were similar in both groups, but there was a trend towards more never-smokers among patients with a long total delay (Table 1). Patients with a total delay of more than two years were more frequently treated with inhalation therapy prior to their diagnosis but without a marked increase in airway obstruction, defined by a FEV_1_/FVC ratio below 0.7 (Table 1). Patients with a long delay also tended to have a higher SGRQ total score and more frequently had a UIP pattern on the diagnostic HRCT, which however did not reach statistical significance (Table 2). Although rarely performed, transbronchial cryobiopsies were more common in patients with a short delay (Table 2). We were unable to calculate total delay for 14 patients, due to imprecise data about their onset of symptoms. Baseline data for this group is presented in the Additional file 1.

**Table 1:** Baseline characteristics of participants with a delay above or below 2 years

	All patients (n = 204)	Total delay > 2 years (n = 98)	Total delay < 2 years (n = 92)	*P*-value
Age (years), mean (SD)	73.7 (7.8)	73.2 (8.0)	73.5 (7.6)	0.79
Sex				0.79
Male, n (%)	158 (77.5%)	74 (75.5%)	71 (77.2%)	
Female, n (%)	46 (22.5%)	24 (24.5%)	21 (22.8%)	
Smoking status				0.28
Never, n (%)	52 (25.6%)	31 (31.6%)	20 (21.7%)	
Active, n (%)	14 (6.9%)	6 (6.1%)	8 (8.7%)	
Former, n (%)	137 (67.5%)	61 (62.2%)	64 (69.6%)	
Pack-years, median (IQR)	25.0 (11.8–40.0)	26.5 (10.9–40.0)	20.0 (10.8–41.7)	0.68
BMI (kg/m^2^), mean (SD)	27.4 (4.6)	28.0 (4.4)	26.8 (4.9)	0.08
Education				0.67
No higher education, n (%)	101 (54.9%)	52 (56.5%)	47 (53.4%)	
Higher education, n (%)	83 (45.1%)	40 (43.5%)	41 (46.6%)	
Previous use of inhalation therapy				< 0.01
No, n (%)	143 (70.1%)	59 (60.2%)	76 (82.6%)	
Yes, n (%)	61 (29.9%)	39 (39.8%)	16 (17.4%)	
Airway obstruction at baseline				0.75
No, n (%)	179 (88.2%)	87 (88.8%)	83 (90.2%)	
Yes, n (%)	24 (11.8%)	11 (11.2%)	9 (9.8%)	
FVC (l), mean (SD)	3.0 (0.8)	3.0 (0.8)	3.1 (0.9)	0.60
FVC (% pred.), mean (SD)	88.9 (19.0)	87.9 (16.8)	91.1 (21.6)	0.25
DLCO (% pred.), mean (SD)	52.6 (13.6)	52.6 (13.6)	53.0 (13.5)	0.84
6MWT-distance (m), mean (SD)	441.7 (106.5)	444.8 (108.1)	448.3 (93.2)	0.82
SaO2 at rest (%), mean (SD)	96.2 (1.9)	96.3 (1.8)	96.2 (1.9)	0.82
SaO2 after 6MWT (%), mean (SD)	88.2 (7.7)	88.2 (7.0)	88.3 (8.2)	0.92
SGRQ total score, mean (SD)	39.1 (19.6)	41.4 (17.8)	37.2 (21.4)	0.20

*SD* standard deviation, *IQR* interquartile range, *FVC* forced vital capacity, *DLCO* diffusion capacity for carbon monoxide, *6MWT* six-minute walk test, *SGRQ* St. George’s Respiratory Questionnaire

Total delay could not be calculated for 14 patients due to the lack of data about onset of symptoms (baseline data of these patients are listed in the Additional file). Airway obstruction was defined as FEV1/FVC <  0.7

**Table 2:** Diagnostic procedures performed for the IPF diagnosis

	All patients (n = 204)	Total delay > 2 years (n = 98)	Total delay < 2 years (n = 92)	*P*-value
HRCT pattern				0.27
UIP, n (%)	142 (73.2%)	75 (78.1%)	59 (67.8%)	
Possible UIP, n (%)	39 (20.1%)	16 (16.7%)	20 (23.0%)	
Not UIP, n (%)	13 (6.7%)	5 (5.2%)	8 (9.2%)	
BAL performed				
No, n (%)	120 (58.8%)	60 (61.2%)	49 (53.3%)	0.27
Yes, n (%)	84 (41.2%)	38 (38.8%)	43 (46.7%)	
Surgical lung biopsy performed				0.25
No, n (%)	180 (88.2%)	83 (84.7%)	83 (90.2%)	
Yes, n (%)	24 (11.8%)	15 (15.3%)	9 (9.8%)	
Cryobiopsy performed				0.02
No, n (%)	188 (92.2%)	95 (96.9%)	81 (88.0%)	
Yes, n (%)	16 (7.8%)	3 (3.1%)	11 (12.0%)	
Velcro crackles on lung auscultation				0.32
No, n (%)	26 (13.6%)	13 (14.1%)	8 (9.3%)	
Yes, n (%)	165 (86.4%)	79 (85.9%)	78 (90.7%)	

*HRCT* high resolution computed tomography, *UIP* usual interstitial pneumonia, *BAL* Bronchoalveolar lavage

The HRCT pattern is classified according to 2011 ATS/ERS/JRS/ALAT guidelines [12]

### Referral pattern

Forty (20%) patients reported three or more visits to their general practitioner before being referred further to a secondary care hospital. When referred to the ILD centres, the majority of patients had been investigated at other community hospitals (*n* = 169, 83%), and only rarely were referred directly from a private respiratory physician (*n* = 6, 3%) or a general practitioner (*n* = 29, 14%). The IPF diagnosis was already suggested at the referring community hospital in 18 (9%) patients before referral.

Most patients from community hospitals were referred from a department of respiratory medicine (*n* = 133, 65%). Fewer patients were referred from departments of internal medicine (*n* = 17, 8%), cardiology (*n* = 5, 2%), emergency departments (*n* = 3, 1%) or other departments (*n* = 10, 5%).

We were able to calculate a total incidence of 2.9 IPF patients per 100,000 persons per year for the eastern part of the country (served by one of the participating ILD centres) where complete data were available. Data on incident IPF patients who were not included in the cohort were not available from other centres and these could thus not be used in estimating the incidence (see Additional file 1 for details).

### Diagnostic delay

**Fig. 2:**
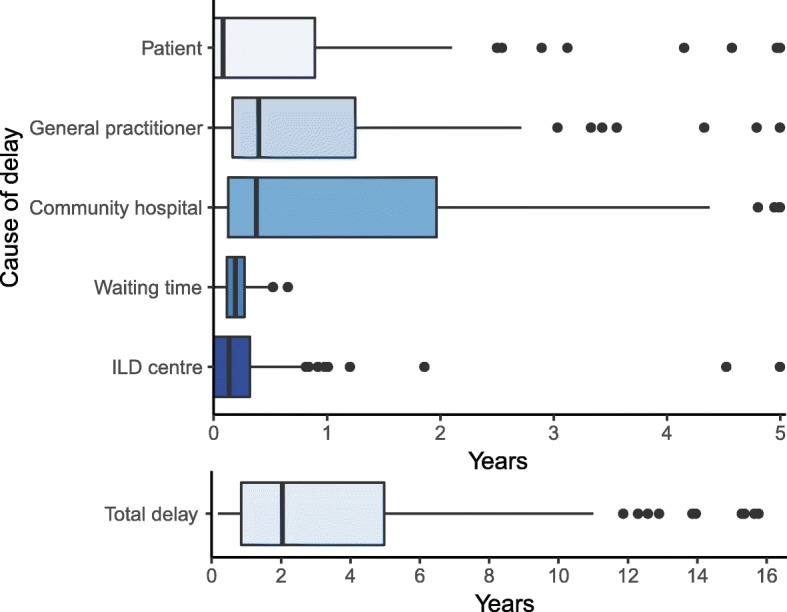
Duration (median, IQR) of total and specific delays due to patient, general practitioner, community hospitals, waiting time and ILD centres. Time periods of the specialized delays are truncated at 5 years to increase legibility. Note different time scales

The median total diagnostic delay was 2.1 years (IQR: 0.9–5.0) and was stable during the study period (see Additional file 1). The delay was mainly attributable to:

1. The patients (onset of symptoms until the first healthcare contact) with a median delay of 0.1 years (IQR: 0–0.9) (Fig. 2).
2. The general practitioners (first contact with a general practitioner until referral to secondary healthcare) with a median delay of 0.4 years (IQR: 0.2–1.2) (Fig. 2).
3. The community hospitals (first visit due to the current respiratory symptoms until referral to an ILD centre) with a median delay of 0.4 years (IQR: 0.1–1.9) (Fig. 2).

The delay due to waiting time (referral to an ILD centre until the first patient visit at this hospital) was 0.2 years (IQR: 0.1–0.3) and the specialized diagnostic workup (first visit at the ILD centre until the final diagnosis) was 0.1 years (IQR: 0–0.3) (Fig. 2). As the diagnostic HRCT scan typically was performed during the waiting time, the median time from the HRCT scan until the final diagnosis was 0.3 years (IQR: 0.1–0.4). Due to highly skewed data of all specific delays, the median total delay was longer than the sum of all specific median delays (Fig. 2).

### Risk factors for diagnostic delay

**Fig. 3:**
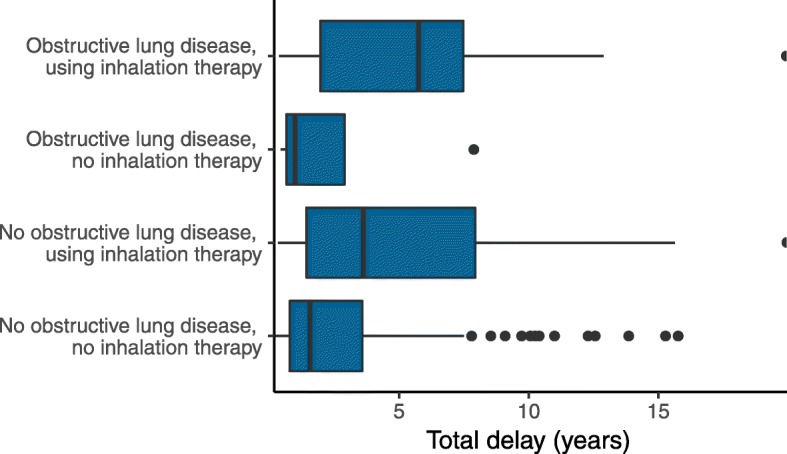
Length of total delay in participants stratified according to obstructive lung disease (asthma or COPD) and previous use of inhalation therapy

In multivariate negative binomial regression analysis, patient delays were prolonged in males and previous inhalation therapy users. Healthcare delays were prolonged in older patients and previous inhalation therapy users. The combined total delay was prolonged in previous inhalation therapy users but not influenced by sex, age or proven obstructive lung disease (Table 3, Fig. 3).

**Table 3:** Incidence rate ratio (IRR) of several risk factors for patient delay, healthcare delay and total delay, assessed by multivariate negative binomial regression

	Patient delay			Healthcare delay			Total delay		
	IRR	95% CI	*p*-value	IRR	95% CI	*p*-value	IRR	95% CI	*p*-value
Patient characteristics									
Age	0.97	0.92–1.02	0.24	1.03	1.01–1.06	0.004*	1.01	0.98–1.03	0.59
Male sex	3.84	1.17–11.36	0.006*	1.01	0.68–1.49	0.95	0.99	0.66–1.48	0.97
Ever smokers	1.34	0.48–3.35	0.51	0.78	0.54–1.11	0.18	0.79	0.54–1.14	0.19
Higher education	2.16	0.91–5.18	0.06	1.28	0.91–1.81	0.14	1.15	0.83–1.60	0.39
Previous use of inhalation therapy	4.68	1.77–13.37	0.0004*	1.98	1.38–2.90	< 0.0001*	1.99	1.40–2.88	< 0.0001*
Clinical findings at diagnosis									
DLCO (%)	1.05	1.02–1.08	0.005*	1.02	1.01–1.03	0.006*	1.02	1.00–1.03	0.02*
FVC (%)	1.02	0.99–1.05	0.06	1.00	0.99–1.01	0.96	1.00	0.99–1.01	0.67
Airway obstruction	1.10	0.23–3.86	0.89	1.57	0.86–2.66	0.11	1.61	0.94–2.61	0.07
SGRQ total score	1.03	1.01–1.07	0.004*	1.01	1.00–1.03	0.003*	1.02	1.01–1.03	0.001*
UIP pattern on HRCT	0.84	0.30–2.11	0.71	1.32	0.89–1.95	0.16	1.47	1.01–2.11	0.04*

*CI* confidence interval, *DLCO* diffusion capacity for carbon monoxide, *6MWT* six-minute walk test, *SGRQ* St. George’s Respiratory Questionnaire, *HRCT* high resolution computed tomography, *UIP* usual interstitial pneumonia *p-value < 0.05

Values of DLCO, FVC and SGRQ-score are obtained at the time of diagnosis. Airway obstruction is defined as FEV1/FVC < 0.7

Clinical findings at the time of diagnosis were also associated with the diagnostic delay (Table 3). Longer patient delays and healthcare delays were associated with preserved DLCO and high SGRQ total score at the time of diagnosis. Consequently, longer total delays were also associated with preserved DLCO, high SGRQ total score but also a usual interstitial pneumonia (UIP) pattern on HRCT at the time of diagnosis (Table 3).

### Alternative diagnoses and treatments

Misdiagnosis was common with respiratory symptoms being attributed to at least one alternative diagnosis in 82 (41%) participants before an IPF diagnosis was established (Table 4). Of these, 62 had received treatment for the alternative diagnosis for a median duration of 7.0 months. Heart disease was the most common misdiagnosis (*n* = 25, 12%) followed by obstructive lung disease (Table 4). However, of the 61 participants with a history of inhalation therapy use, only 20 (33%) fulfilled the diagnostic criteria for either asthma or COPD. Treatment with inhalation therapy increased the total diagnostic delay in patients with and without proven obstructive lung disease (Fig. 3).

**Table 4:** Previous diagnosis and treatment before an IPF diagnosis was made

	All patients (n = 204)
Alternative diagnoses for respiratory symptoms	
Heart disease	25 (12.3%)
Asthma	17 (8.3%)
Chronic bronchitis	11 (5.4%)
COPD	10 (4.9%)
Emphysema	3 (1.5%)
Other	18 (8.8%)
Reports of pneumonia during the previous 2 years before IPF diagnosis	
0	88 (49.2%)
1	32 (17.9%)
2	28 (15.6%)
3 or more	31 (18.0%)
Antibiotic courses for pneumonia during the previous 2 years before IPF diagnosis	
0	91 (50.6%)
1	35 (19.4%)
2	31 (17.2%)
3 or more	23 (11.3%)
Previous medical treatments	
Inhalation therapy	
SABA	39 (19.1%)
LABA	29 (14.2%)
LAMA	21 (10.3%)
ICS	26 (12.7%)
Prednisolone	34 (16.7%)
Other immunosuppressive treatment^a^	5 (2.5%)
Proton pump inhibitor or H2-receptor antagonist	85 (41.7%)
Antacids	57 (31.7%)

*SABA* short acting beta agonist, *LABA* long acting beta agonist, *LAMA* long acting muscarinic antagonist, *ICS* inhaled corticosteroid. ^a^Azathioprine (n = 2), mycophenolate mofetil, oral budesonide and cyclophosphamide (each n = 1)

A total of 51% of patients reported at least one occurrence of pneumonia during the two years before their IPF diagnosis, while 49% received treatment for at least one instance of pneumonia (Table 4).

Thirty-five (18%) of all participants had received at least one prescription of prednisolone or other immunosuppressive treatment before referral. A large proportion of patients (42%) had received a prescription of proton pump inhibitor (PPI) and 32% also reported taking over-the-counter antacid therapy (Table 4). None of the patients were treated with specific antifibrotic treatment until they received their diagnosis of IPF at the ILD centres.

## Discussion

We studied the referral pattern and diagnostic delay in a cohort of IPF patients and found that it was mainly attributable to patients, general practitioners and community hospitals. Previous inhalation therapy use was a major risk factor for a delayed diagnosis. Male sex was associated with a prolonged patient delay and older age was associated with a prolonged healthcare delay. Patients were often misdiagnosed and treated before a final diagnosis of IPF was made.

We present for the first time a detailed description of IPF patients’ path towards the IPF diagnosis and the different components of the diagnostic delay in incident patients and thus with limited recall bias. We highlight the three main sources of delay: patients themselves, general practitioners and community hospitals (most commonly departments of respiratory medicine). Future efforts to reduce the diagnostic delay should be directed at these three sources which could be achieved by further increasing awareness of IPF among patients, general practitioners and hospital physicians (both pulmonologists and other specialists). However, while the delay at ILD centres often was shorter, it still contributed to the total delay in many patients and the median time from the diagnostic HRCT until the final diagnosis was still 0.3 years. Therefore, there is a potential for interventions to shorten the delay at all steps towards the IPF diagnosis, which should be further explored. A delay, however, is not always the result of a mistake or missed diagnosis. It is possible that some patients were delayed because they did not fulfil diagnostic criteria for IPF during the early stages of their disease, which is a recognized limitation of the 2011 ATS/ERS/JRS/ALAT guidelines [16].

We expected a shortened diagnostic delay due to an increased awareness of IPF during recent years which unfortunately could not be confirmed. The availability of pharmacological treatments during the past few years resulted in an increase in the number of patients referred to ILD centres, but this increase has so far failed to impact the diagnostic delay in IPF [7]. We found a median total diagnostic delay of 2.1 years, which is similar to previous reports of IPF patients and general ILD populations, despite new treatment options, new diagnostic guidelines and increased awareness of IPF [4–8, 17–20].

In addition to the median length of the diagnostic delay of 2.1 years, we found a high spread of the specific and total delays with 25% of patients having a delay of more than 5 years. This finding contributes to the evolving understanding that some patients with IPF can have a slowly progressive phenotype [1]. However, some patients reported very long delays of up to 20 years, which are unlikely to be due to symptoms caused by IPF. Rather, the patient-reported symptoms could be caused by an alternative disease, such as cardiovascular disease or COPD, which are common comorbidities in patients with IPF [21]. In addition, recall bias can affect the patients’ ability to remember important information about the onset of their symptoms. However, the specific healthcare delays are defined by objective data from electronic records and support the hypothesis of a phenotype with slowly progressive IPF. The possible identification of interstitial lung abnormalities on lung CT many years prior to the development of clinical interstitial lung disease further contributes to this observation [22–24].

A first step to reduce the diagnostic delay is to identify risk factors for a delay and recognize target groups for interventions. We are unaware of previous studies providing estimates of risk factors for a delayed diagnosis of IPF. Interestingly, the risk factors for patient delay differ from those for healthcare delay. The previous use of inhalation therapy and male sex were risk factors for a prolonged patient delay. The increased delay in previous inhalation therapy users could be attributable to treatment attempts with inhalation therapy for unexplained respiratory symptoms in the primary care sector, which has been suggested previously [12, 18]. Airway obstruction was not a risk factor for a diagnostic delay, which indicates frequent futile treatment attempts with inhalation therapy in patients without obstructive lung disease. However, it is possible that an obstructive lung disease was masked by a concomitant reduction in FVC in some patients. While treatment attempts with inhalation therapy can be warranted in patients with unexplained respiratory symptoms, these patients should be closely monitored and referred for further workup when symptoms do not improve. In addition, a correctly performed and interpreted spirometry could help to quickly rule out airway obstruction as a cause of breathlessness in these patients. Improved diagnostic tools, including screening or effective diagnostic biomarkers, would make the diagnosis more accessible, also for non-ILD specialists, and are desperately needed.

Patients with a prolonged delay had relatively preserved DLCO at the time of diagnosis, suggesting that patients with more severe IPF were quickly diagnosed. On the other hand, patients with a prolonged delay reported a high symptom burden as indicated by a higher SGRQ total score. This apparent discrepancy could be explained by the complexity of quality of life data, which can be impacted by many factors, including a prolonged diagnostic delay but also by comorbidities, misdiagnosis, treatment and multiple healthcare contacts. Besides, the SGRQ total score has limitations in quantifying disease severity in IPF patients [25].

Patients reported frequent misdiagnosis prior to their final IPF diagnosis, which is in line with the presented risk factors for a diagnostic delay, and with previous patient surveys [12, 17, 20]. The frequent treatment with inhalation therapy for obstructive lung disease, despite the lack of airway obstruction, confirms this problem. In addition, the previous treatments were often given for long periods (median duration of treatment was 7.0 months) and included potentially harmful treatments such as prednisolone and other immunosuppressive drugs, which are often prescribed for other fibrotic lung disease such as chronic hypersensitivity pneumonitis, a common differential diagnosis to IPF. The frequent occurrences of pneumonia in the two years prior to the IPF diagnosis could either be due to an increased risk of infection in patients or misinterpretation of crackles on auscultation. Screening for finger clubbing and velcro crackles on auscultation, particularly if they do not resolve after treatment of suspected pneumonia, should prompt further diagnostic evaluation [26]. A treatment attempt for any suspected disease prolongs the diagnostic delay, and most importantly delays the initiation of effective antifibrotic treatment. Early referral of patients with an uncertain diagnosis to a centre with expertise in ILD is essential and could contribute to shortening the diagnostic delay.

Based on data from one participating centre, we could estimate an annual incidence of 2.9 IPF patients per 100,000 inhabitants per year. However, it might be suspected that some older and frail patients, or patients with severe comorbidities, were not referred for a diagnostic workup at an ILD centre. Our calculated incidence is in the lower end of the range of other estimates (0.6–17.4 cases of IPF per 100,000 inhabitants per year) which suggests a continuing underdiagnosis of IPF [27]. Previous reports have often been performed retrospectively or are based on registries and insurance claims (which can overestimate incidence due to miscoding) and many estimates predate the current diagnostic guidelines [27–29]. In contrast to several previous studies, all patients included in our cohort had a confident IPF diagnosis confirmed at a specialised ILD centre. A Danish study estimated the IPF incidence in 2003–2009 to be 1.3 patients per 100,000 inhabitants per year [7]. Thus, despite the apparent underdiagnosis, we can confirm a general increase in the incidence of IPF diagnosis in a comparable setting.

Our study has several strengths but also some limitations. A major strength is the prospective inclusion of a large part of all incident IPF patients in two ILD centres in the region increasing the generalizability of the results. In addition, we included patients immediately after their IPF diagnoses, thus reducing recall bias of important information, such as the date of symptom onset. However, as most patients experienced a diagnostic delay of several years, there remains a risk of recall bias in our cohort. Wherever possible, time points were extracted from electronic medical records, resulting in more reliable data compared with surveys with patient reported data. However, some data were still patient reported, and thus not entirely objective. This includes the date of symptom onset and the first healthcare contact. These data are inherently subjective and could not have been collected in a different manner. The definition of symptom onset can also be debated. Many patients with IPF have comorbidities which could give respiratory symptoms (i.e., cardiovascular disease or COPD) making it difficult to define the onset of symptoms caused by IPF. However, proven obstructive pulmonary disease was not a risk factor for a prolonged delay.

Our study was performed in one single country and findings cannot simply be generalized to other countries and healthcare systems. While treatment of IPF in Denmark is centralised to a few ILD centres, it may not be in other countries, potentially leading to more missed or delayed diagnoses. Also, the organization and responsibilities of primary, secondary and tertiary care differ between countries. Due to these differences, we considered the combined healthcare delay in the statistical analyses, rather than the specific delays (general practitioner, community hospital or ILD centre). Nevertheless, we believe that patients with IPF in other health systems will meet the same obstacles on their path towards a diagnosis of IPF, due to the rarity of the condition and the difficulty in establishing a confident diagnosis.

## Conclusion

In conclusion, this study has shown that diagnostic delay in IPF is mainly attributable to patients, general practitioners and community hospitals. Patients were often misdiagnosed and treated before a final diagnosis of IPF was made. Previous inhalation therapy use was a major risk factor for a delayed diagnosis, and male sex and older age were risk factors for patient delay and healthcare delay respectively. Efforts to reduce the diagnostic delay should focus on these risk factors.

## Additional file

Additional file 1: **Figure S1.** Recruitment and follow-up of participants in the PFBIO cohort. Centre A: Gentofte Hospital, Centre B: Aarhus University Hospital. **Figure S2.** Diagnostic delay length in all participants where the total delay was possible to calculate (*n* = 190). **Table S1.** Baseline data of participants without a calculated total delay available for analysis. (DOCX 73 kb)

## Abbreviations

6MWT: 6-min walk test
COPD: Chronic obstructive pulmonary disease
DLCO: Diffusing capacity for carbon monoxide
HRCT: High Resolution computed tomography
ILD: Interstitial lung disease
IPF: Idiopathic pulmonary fibrosis
IQR: Interquartile range
PPI: Proton pump inhibitor
SGRQ: St. George’s respiratory questionnaire
UIP: Usual interstitial pneumonia
