# Supplementary material for: Risk factors for diagnostic delay in idiopathic pulmonary fibrosis
Source: Respir Res. 2019 May 24;20:103. doi: 10.1186/s12931-019-1076-0 (PMC6534848; doi:10.1186/s12931-019-1076-0)
Supplement: Supplementary file 1 — Figure S1. Recruitment and follow-up of participants in the PFBIO cohort. Centre A: Gentofte Hospital, Centre B: Aarhus University Hospital. Figure S2. Diagnostic delay length in all participants where the total delay was possible to calculate (n = 190). Table S1. Baseline data of participants without a calculated total delay available for analysis. (DOCX 73 kb) [file 12931_2019_1076_MOESM1_ESM.docx]

## Supplementary material

### Recruitment

Two hundred and four incident IPF patients were included in the analysis. A total of 100 patients were referred from hospitals in the Capital Region of Denmark (1.8 included patients per 100,000 inhabitants per year), 45 were referred from hospitals in Region Zealand (1.8 included patients per 100,000 inhabitants per year), 21 were included from hospitals in the Central Denmark Region (0.8 included patients per 100,000 inhabitants per year of active recruitment), three were referred from a hospital on the Faroe Islands, 6 were referred from private respiratory physicians and 29 were referred from general practitioners. 41 patients were diagnosed with IPF but not included in the cohort with the reasons for non-participation listed in supplementary Figure 1.


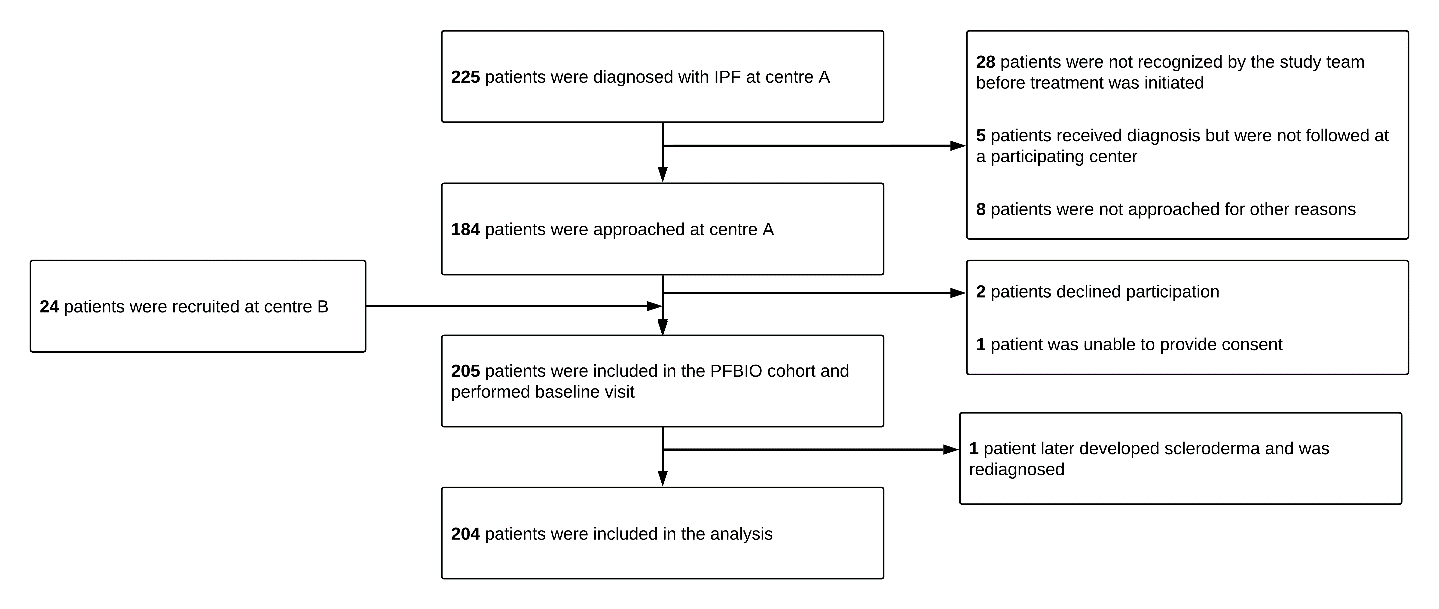


**Supplementary Figure 1:** Recruitment and follow-up of participants in the PFBIO cohort. Centre A: Gentofte Hospital, Centre B: Aarhus University Hospital

Population of eastern Denmark (Capital Region and Region Zeeland), served by Centre A: 2 640 000

Recruitment period of Centre A: 2.9 years


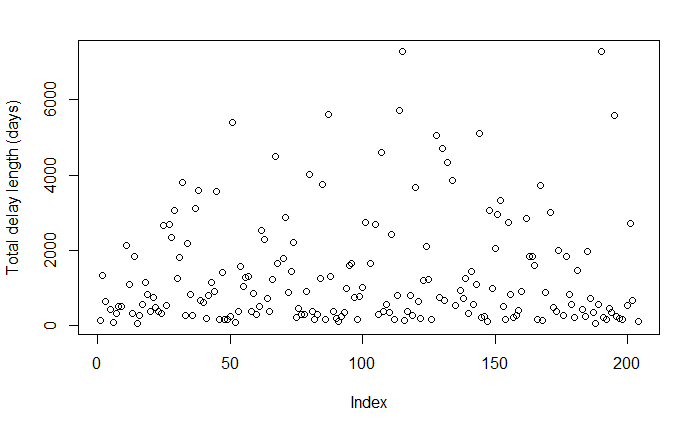


**Supplementary Figure 2:** Diagnostic delay length in all participants where the total delay was possible to calculate (n = 190)

### Baseline characteristics

|  | **Total delay possible to calculate (n=190)** | **Total delay not possible to calculate (n=14)** | **Overall (N=204)** |
| --- | --- | --- | --- |
| Age, mean (SD) | 73.4 (7.8) | 77.8 (6.0) | 73.7 (7.8) |
| Sex |  |  |  |
| Male | 145 (76.3%) | 13 (92.9%) | 158 (77.5%) |
| Female | 45 (23.7%) | 1 (7.1%) | 46 (22.5%) |
| Smoking status |  |  |  |
| Never | 51 (26.8%) | 1 (7.7%) | 52 (25.6%) |
| Active | 14 (7.4%) | 0 (0.0%) | 14 (6.9%) |
| Former | 125 (65.8%) | 12 (92.3%) | 137 (67.5%) |
| Pack-years, median (IQR) | 25.0 (10.6–40.0) | 28.5 (16.3–52.5) | 25.0 (11.8–40.0) |
| BMI, mean (SD) | 27.4 (4.6) | 27.1 (4.8) | 27.4 (4.6) |
| Education |  |  |  |
| No higher education | 99 (55.0%) | 2 (50.0%) | 101 (54.9%) |
| Higher education | 81 (45.0%) | 2 (50.0%) | 83 (45.1%) |
| Previous use of inhalation therapy |  |  |  |
| No | 135 (71.1%) | 8 (57.1%) | 143 (70.1%) |
| Yes | 55 (28.9%) | 6 (42.9%) | 61 (29.9%) |
| Airway obstruction at baseline |  |  |  |
| No | 170 (89.5%) | 9 (69.2%) | 179 (88.2%) |
| Yes | 20 (10.5%) | 4 (30.8%) | 24 (11.8%) |
| FVC (l), mean (SD) | 3.1 (0.9) | 2.8 (0.6) | 3.0 (0.8) |
| FVC (% pred.), mean (SD) | 89.5 (19.3) | 80.5 (13.7) | 88.9 (19.0) |
| DLCO (% pred.), mean (SD) | 52.8 (13.5) | 49.4 (15.8) | 52.6 (13.6) |
| 6MWT-distance (m), mean (SD) | 446.4 (101.0) | 375.5 (156.3) | 441.7 (106.5) |
| SaO2 at rest (%), mean (SD) | 96.2 (1.9) | 96.2 (1.8) | 96.2 (1.9) |
| SaO2 after 6MWT (%), mean (SD) | 88.2 (7.6) | 87.5 (9.0) | 88.2 (7.7) |
| SGRQ total score, mean (SD) | 39.3 (19.7) | 34.2 (12.0) | 39.1 (19.6) |
| HRCT-pattern |  |  |  |
| UIP | 134 (73.2%) | 8 (72.7%) | 142 (73.2%) |
| Possible UIP | 36 (19.7%) | 3 (27.3%) | 39 (20.1%) |
| Not UIP | 13 (7.1%) | 0 (0.0%) | 13 (6.7%) |
|  |  |  |  |
| **Supplementary Table 1:** Baseline data of participants without a calculated total delay available for analysis | | | |

Time from diagnostic CT-scan to diagnosis (median): 0.3 years (IQR: 0.1-0.4)

### Baseline questionnaire

The following questionnaire about the time from symptoms onset until the final diagnosis of IPF was answered by participants at the baseline visit. The questions were part of a more detailed questionnaire and patients were informed that they were asked about their path towards the IPF diagnosis. If there were any questions or uncertain answers, the questionnaire was followed up by an interview.

If the patient could remember a time period (i.e. June to July 2007 or November 2018) rather than the specific date, the median date in this period was used for calculating delays (July 1, 2007 or November 15, 2018).

The questions related to this article have been translated from Danish and are presented below.

| The time before you received a diagnosis of pulmonary fibrosis |
| --- |
| **Which symptoms initially made you contact a doctor?**  **Choose one or more**   - Dry cough - Productive cough - Coughing up blood - Shortness of breath - Chest pain - Uncomfort in the chest - Respiratory sounds - Fever - Loss of energy or tiredness - Loss of weight - Other. What? _______________________________________ |
| **When did you for the first time notice these symptoms? Please write as precisely as you can remember (preferably date or month).**  _________________________________________ |
| **When did you for the first time contact a doctor with these symptoms? Please write as precisely as you can remember (preferably date or month).**  __________________________________________ |
| **How many times have you had pneumonia, bronchitis or a prolonged cold during the last two years?**  Approximately _______________ times |
| **How many times have you received antibiotics against pneumonia, bronchitis or a prolonged cold during the last two years?**  Approximately _______________ times |
| **Did your general practitioner or the referring hospital arrange for a chest x-ray or a CT scan before you were referred to our department of respiratory disease?**   - X-ray - CT scan - No |
| **Did your general practitioner or the referring hospital perform pulmonary function tests before you were referred to our department of respiratory disease?**   - Yes. When? _______________________________________ - No |
| **How many times did you visit your general practitioner before being referred to a hospital?**  _______ times |
| **Where you seen by other hospital doctors for your respiratory symptoms before you were referred to our department of respiratory disease?**   - Yes - No |
| **If you were seen by other hospital doctors, at which kind of department was it?**  **Choose one or more**   - Emergency department - Department of internal medicine - Department of respiratory medicine - Department of cardiology - Department of surgery - Other. Which? _______________________________________ |
| **Did you receive alternative diagnoses for your respiratory symptoms before you were diagnosed with pulmonary fibrosis?**  **Choose one or more**   - Bronchitis - Pneumonia - Asthma - COPD - Emphysema - Heart disease - Other. What? _______________________________________ - No |
| **Were you treated for any of these diseases?**   - Yes. How? __________________________ How long? ____________________________ - No |
| **If you received any of these alternative diagnoses, when did you receive it? Please write as precisely as you can remember (preferably date or month).**  _______________________________________________________ |
| **Which of the following describes your view of this alternative diagnosis?**  **Choose one**   - The alternative diagnosis is correct. It contributes to my current symptoms. - The alternative diagnosis is correct. I do have this disease but it is not contributing to my current symptoms. - The alternative diagnosis is wrong. I do not have this disease. |
